# Intrinsic Thermal conductivities of monolayer transition metal dichalcogenides MX_2_ (M = Mo, W; X = S, Se, Te)

**DOI:** 10.1038/s41598-019-40882-2

**Published:** 2019-03-14

**Authors:** Muhammad Zulfiqar, Yinchang Zhao, Geng Li, ZhengCao Li, Jun Ni

**Affiliations:** 1 State Key Laboratory of Low-Dimensional Quantum Physics, Department of Physics, Tsinghua University, Beijing, 100084 People’s Republic of China; 2 Collaborative Innovation Center of Quantum Matter, Beijing, 100084 People’s Republic of China; 3 Department of Physics, University of Sargodha, 40100 Sargodha, Pakistan; 4 Department of Physics, Yantai University, Yantai, 264005 People’s Republic of China; 5 Department of Material Science and Engineering, Tsinghua University, Beijing, 100084 People’s Republic of China

## Abstract

The successful synthesis of the single to few layer transition metal dichalcogenides has opened a new era in the nanoelectronics. For their efficient implementations in the electronic devices while taking care of their overheating issues, the characterization of their thermal transport properties is extremely vital. So, we have systematically investigated the thermal transport properties of monolayer transition metal dichalcogenides MX_2_ (M = Mo, W; X = S, Se, Te) by combining the first-principles calculations with Boltzmann transport equation. We find that monolayer WTe_2_ possesses the lowest lattice thermal conductivity *κ*_*L*_ (33:66 Wm^−1^K^−1^ at 300 K) among these six semiconducting materials, in contrast to the highest *κ*_*L*_ (113:97 Wm^−1^K^−1^ at 300 K) of WS_2_ among them. Further analyses reveal that the higher (lower) anharmonic and isotopic scatterings together with the lower (higher) phonon group velocities lead to the lowest (highest) value of *κ*_*L*_ in WTe_2_ (WS_2_) monolayer. In addition, we have also calculated the cumulative thermal conductivity *κ*_*C*_ as a function of mean free path, which indicates that the nanostructures with the length of about 400 nm would reduce *κ*_*L*_ drastically. These results offer important understanding from thermal conductivity point of view to design the 2D transition metal dichalcogenides MX_2_ (M = Mo, W; X = S, Se, Te) electronics.

## Introduction

In the past few years, two-dimensional (2D) materials like graphene have attracted great attention owing to the unique properties related to low dimensionality^1^. With the boom of 2D materials, monolayer transition metal dichalcogenides (TMDC) MX_2_ monolayers have emerged as new terrace for exploring 2D semiconducting physics, which facilitates the studies such as photodetectors, transistors, and electroluminescent devices^2^. For instance, due to the band gap of 1.0–2.0 eV, TMDC MX_2_ monolayer field-effect transistors are expected to possess high on/off ratio^3,4^. It has been predicted that an indirect bandgap can be changed into the direct one as the bulk TMDC MX_2_ crystal become thinned to the monolayers, which provides more possibilities to manipulate the electronic dispersion of TMDC MX_2_ monolayers at nanoscale^5^. Furthermore, for the TMDC MX_2_ monolayers, the biaxial stain can adjust the bandgap reversibly, which makes the system capture an increased range of solar spectrum^6^. In addition, the TMDC MX_2_ monolayers are also good candidates for spintronics and valleytronics researches^7,8^. In all of these applications, thermal properties have significant influences on the performance of the devices. Based on the conditions of specific application, different thermal conductivities *κ* are needed. As an example, a high *κ* is required for efficient heat dissipation in the high-performance electronic devices, while a low *κ* is preferred in thermoelectric application. On the bases of the phonon Boltzmann transport equation, the calculated thermal conductivities at room temperature for WS_2_, MoS_2_ and MoSe_2_ monolayers are 142, 103, and 54 Wm^−1^ K^−1^, respectively^9^. On the other hand, the experimentally measured thermal conductivities for the MoS_2_ (34.5 ± 4 Wm^−1^ K^−1^)^10^ and WS_2_ (32 Wm^−1^ K^−1^)^11^ monolayers differ from the theoretical predictions. Also, much lower room temperature thermal conductivity (23.2 Wm^−1^ K^−1^) has been proposed for monolayer MoS_2_ as compared to graphene from first-principles calculations combined with the nonequilibrium Green’s function calculations^12^. Furthermore, sulfur vacancies and molecular adsorption can modulate the carrier densities which in turn greatly influence the Seebeck coefficient in case of MoS_2_ monolayer^13^. The phonon transport is greatly affected by the crystal structure, atomic mass, interatomic bonding, and anharmonicity^14–16^. Recently, theoretical investigations have been performed to elaborate the role of mass, structure, bond strength, and anharmonicity in thermal expansion as well as thermo-mechanics of TMDC MX_2_ monolayers and other 2D materials^17–20^. In contrast to the electronic, optical, and mechanical properties of single-layer or few-layer TMDC, which have been widely studied, the investigations of the thermal properties still lacking. Nevertheless, the significant knowledge of thermal properties is essential regarding the performance and reliability of the nano-devices. According to the classical theory, due to their heavy atom mass and low Debye temperature, the thermal conductivities of TMDC sheets are low^21^. Thus, single-layer or few-layer TMDC sheets are considered as potential thermoelectric materials^22–26^. From some classical molecular dynamics (MD) simulations implemented with empirical inter-atomic potentials, it is reported that the thermal conductivity of the single-layer MoS_2_ is found less than 10 W/mK^27,28^, as compared to the measured thermal conductivities for the single-layer and multilayer MoS_2_ which are usually larger than 30 W/mK^10,29^. Owing to this large variance in the results, so far reported in literature, here we are encouraged to present a detailed study on the intrinsic lattice thermal conductivities of TMDC MX_2_ (M = Mo, W; X = S, Se, Te) monolayers. Even though, the size dependency of ZT and the Seebeck coefficient in case of the MX_2_ (M = Mo, W; X = S, Se, Te) monolayers have been explored for thermoelectric applications. However, the details for the suggested *κ*_*L*_ regarding the TMDC MX_2_ monolayers are lacking. We have try to analyze the uninvestigated details regarding the intrinsic thermal conductivities for considered materials by mutually combining the scattering effects, group velocities, relaxation time, and nanostructuring.

In this paper, we systematic study the thermal transport properties in monolayer TMDC MX_2_ (M = Mo, W; X = S, Se, Te) using first-principles calculations and an iterative solution of the Boltzmann transport equation (BTE) for phonons^30–32^. We find that monolayer WTe_2_ possesses the lowest lattice thermal conductivity *κ*_*L*_ (33.66 Wm^−1^ K^−1^ at 300 K) among these six semiconducting materials. We have analyzed the vital role played by the anharmonic and isotopic scatterings along phonon group velocities due to which WTe_2_ monolayer attained the lowest lattice thermal conductivity *κ*_*L*_. Significantly, the lowest obtained value of *κ*_*L*_ become easy to analyze by combining the group velocities with relaxation time. Moreover, the cumulative thermal conductivity *κ*_*C*_ as a function of mean free path, clearly indicates that nanostructuring up to the length of 400 nm would reduce *κ*_*L*_ drastically which differ from graphene.

## Computational Methods and Structural Modeling

From the solution obtained through Boltzmann transport equation (BTE)^30^, we have calculated thermal transport properties. The equation represents the lattice thermal conductivity measure along the *x* direction i.e.

1 $${\kappa }_{L}^{xx}=\frac{1}{{k}_{B}{T}^{2}{\rm{\Omega }}N}\sum _{\lambda }\,{f}_{0}({f}_{0}+1){(\hslash {\omega }_{\lambda })}^{2}{v}_{g}^{x,\lambda }{F}_{\lambda }^{x},$$

where, *N*, *k*_*B*_, and Ω, represents the density of *q* points in the first Brillouin zone, Boltzmann constant, and the unit cell volume, respectively. *f*_0_ denotes the well-known Bose-Einstein distribution function under equilibrium condition. *ω*_*λ*_ denotes the particular phonon frequency for such phonon mode *λ* which includes both wave vector *q* and phonon branch *ν*. Finally, ℏ represents reduced Planck-constant and ${v}_{g}^{x,\lambda }$ demonstrate the phonon group-velocity for mode *λ* in the x-direction. ${F}_{\lambda }^{x}$ can be obtained from^30^

2 $${F}_{\lambda }^{x}={\tau }_{\lambda }({v}_{g}^{x,\lambda }+{{\rm{\Delta }}}_{\lambda }),$$

Where *τ*_*λ*_ denotes the lifetime calculated with help of phonon relaxation-time approximation i.e. (RTA). To eliminate the inaccuracy of RTA, we have included Δ_*λ*_ which is a correction term that can easily be obtained by solving BTE iteratively. By setting Δ_*λ*_ equal to zero, the value of ${\kappa }_{L}^{xx}$ is obtained from RTA. In the present work, the RTA and the iterative solution (ITS) of the BTE are both used to calculate *κ*_*L*_. To determine *κ*_*L*_, the ShengBTE-code^30^ is utilized. For ShengBTE calculations, the harmonic and anharmonic interatomic force constants i.e. IFCs are obtained with the help of PHONOPY program^33^ joined with VASP^34,35^ and THIRDORDER.PY script provided with ShengBTE. Using finite-difference approach implemented in THIRDORDER.PY, anharmonic IFC3 is calculated using the 4 × 4 × 1 supercell by considering the interaction up to third-nearest neighbors^33^. The isotopic effect is calculated from the equation (10) by considering the natural occurring case, while the size dependence effect is estimated from the cumulative thermal conductivity versus the scalar mean free path defined by the equation (21)^30^. In calculations, ion cores electron are treated by the projector-augmented wave potentials (PAW)^36^, while valance electrons are treated by a plane-wave basis set by using an cutoff energy of 520 eV and the exchange-correlation-functional of generalized-gradient approximation (GGA) of Perdew-Burke-Ernzerhof (PBE) are utilized^37^. The primitive cell is relaxed until the forces experienced by each atom become less than 10^−8^ eV/Å with the energy convergence criteria of 10^−8^ eV. To simulate Brillouin zone, 15 × 15 × 1 Γ-centered Monkhorst-Pack *k*-point mesh is utilized. At last, for ShengBTE calculations, a *q*-mesh of 30 × 30 × 1 is used to simulate the corresponding **q** space integration.

## Results and Discussion

Transition metal dichalcogenides crystallize in hexagonal crystal lattice, resulting in a honeycomb structure. A typical TMDC is built up of X − M − X layers. Each MX_2_ layer consists of a transition metal M atomic plane sandwiched between two chalcogen X atomic planes in a trigonal prismatic arrangement through Van der Waals (vdW) interaction. First, we have fully relaxed the atomic positions as well as lattice vectors in order to obtain the optimized geometry of TMDC MX_2_ (M = Mo and W; X = S, Se, and Te) monolayers. The optimized value of lattice constant *a*_0_ is found to be 3.18 Å (MoS_2_), 3.32 Å (MoSe_2_), 3.55 Å (MoTe_2_), 3.18 Å (WS_2_), 3.31 Å (WSe_2_), and 3.55 Å (WTe_2_). The value of *a*_0_ increases going from S to Se to Te, while it remains the same with the change in transition metal, i.e. *a*_0_(MoX_2_) ≅ *a*_0_ (WX_2_).

### Phononic band structures of monolayer *MX*_2_

**Figure 1:**
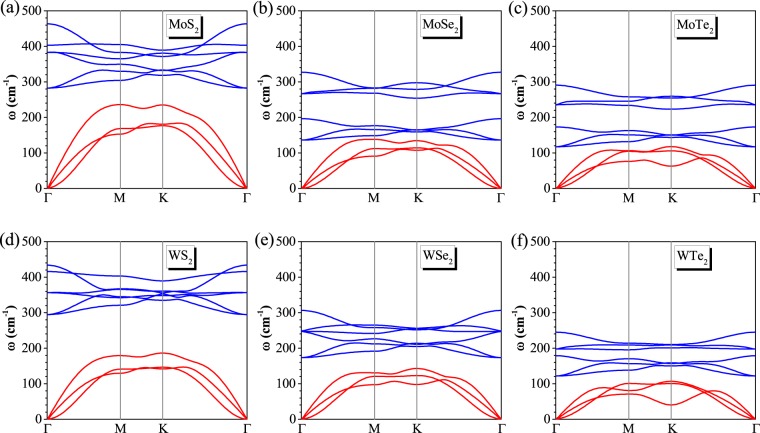
Phonon dispersion for TMDC MX_2_ monolayers. The acoustic and optic branches are presented in red and blue colors.

The phonon dispersion along the high-symmetry Γ − M − K − Γ directions in the first Brillouin zone for the TMDC *MX*_2_ monolayers are plotted in Fig. 1(a–f). As shown in Fig. 1, the acoustic branches for all the *MX*_2_ monolayers have significant differences in the Γ − M and Γ − K directions, particularly near the Brillouin-zone boundary. The unit cell of TMDC *MX*_2_ monolayers have three atoms, resulting in the nine dispersion branches: three acoustic phonon modes and six optical phonon modes. In general, the MTe_2_ phonon bands are significantly shifted down to lower frequencies as compared to the frequencies of the rest of the considered systems. The cause of this trend is the larger mass of the tungsten atoms, and therefore their lower vibration frequency. Noticeably, th general down-shift of the phonon branches associated to the TMDC MX_2_ monolayers gradually reduces the phonon gaps. For instant the acoustic and optical branches are separated by a gap of about 67.95, 10.07, 22.95, 141.06, 58.99, and 37.26 *cm*^−1^ for all of TMDC MX_2_ monolayers, respectively. Moreover, the slopes of the MS_2_(Se_2_) monolayers acoustical phonon bands near zero energy are much steeper than those of WTe_2_ and MTe_2_ monolayers, indicating a higher thermal conductance for them. All of our results are in good overall agreements for already reported works with a little bit discrimination, which supports our methods utilized in these calculations^9,38–40^.

### Intrinsic thermal conductivities *κ*_*L*_

**Figure 2:**
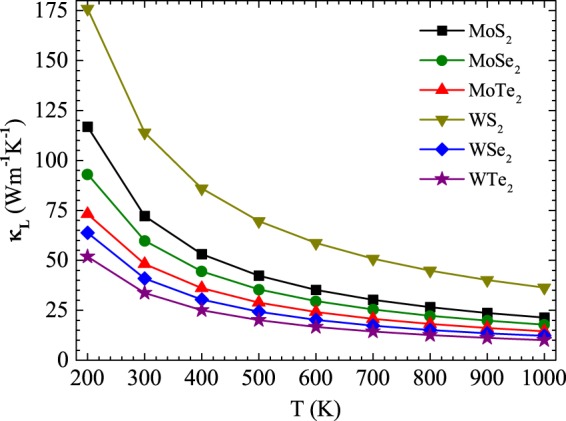
Lattice thermal conductivity as functions of temperature for TMDC MX_2_ monolayers.

The intrinsic thermal conductivities *κ*_*L*_ associated with TMDC MX_2_ monolayers between temperatures 200 and 1000 K are presented in Fig. 2. With reference to Eq. (1), the originally obtained value of *κ*_*L*_ from the BTE calculations needs to be re-sized by a factor of c/h, where c and h denoted the lattice constant along z-axis and the thickness of the monolayer sheets, respectively. The iterative solutions (ITS) of the BTE results shown in Fig. 2 reveal that for all TMDC MX_2_ monolayers, thermal conductivities decrease with increasing temperature due to the phonon domination as found in other materials^41,42^.

The calculated values of *κ*_*L*_ are found to be very sensitive to particular atomic elements, from which the TMDC MX_2_ monolayers comprising of, for all range of temperatures. For instance at 500 K, the ITS (RTA) values of *κ*_*L*_ for WS_2_, MoS_2_, WSe_2_, MoSe_2_, MoTe_2_, and WTe_2_ monolayers are 58.3, 32.2, 18.2, 17.3, 16.3, 13.1 Wm^−1^ K^−1^, respectively. Also, the calculated results are quite dependent on the mass difference between the transition metals and chalcogen atoms, indicating that the mass difference plays a vital role in determining the values of *κ*_*L*_. In Fig. 2, the MoTe_2_ and the WTe_2_ monolayer lies at the bottom because “Te” atoms possess high atomic weights as compared to “S” and “Se” atoms. At 300 K, for WTe_2_ the value of *κ*_*L*_ obtain is 33.66 Wm^−1^ K^−1^, which are lower than the rest of the members of TMDC MX_2_ monolayers.

### Size dependency

**Figure 3:**
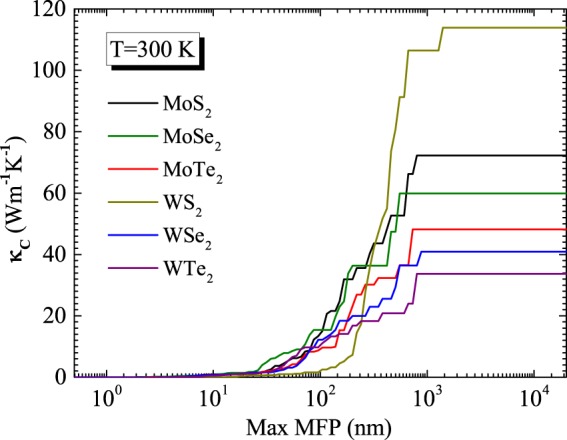
Cumulative thermal conductivity *κ*_*c*_ as a function of phonon maximum mean-free path (MFP) for MX_2_.

To understand the size dependence of the *κ*_*L*_, we have plotted the thermal conductivity accumulation functions for MX_2_ monolayers at a temperature of 300 K in Fig. 3. The accumulation function for *κ*_*L*_ clearly signifies how the phonons with mean free paths contribute to the total thermal conductivity. Phonons with mean free paths extending over two orders of magnitude ranging from 50 nm to 1 *μ*m significantly take part in developing the thermal conductivity in the MX_2_ monolayers. Accumulation function looks like a step function for all MX_2_ monolayers. But the phonon having mean free paths falling between 50 and 500 nm play a vital role for the MX_2_ monolayers except the WTe_2_ monolayer because major contributions for the WTe_2_ monolayer come from the phonons with mean free paths in range 100 to 700 nm. Similar accumulation step function has also been reported for the blue phosphorene and the silicene. Also, the phonons having mean free paths in above mentioned ranges significantly differ from the blue phosphorene and the silicene^43,44^. Strikingly, as can be noticed from Fig. 3, changing the sample size greater than 10 nm exhibits negligible effect on the thermal conductivity of MX_2_ monolayers at a temperature 300 K.

### Relaxation time for three-phonon processes

**Figure 4:**
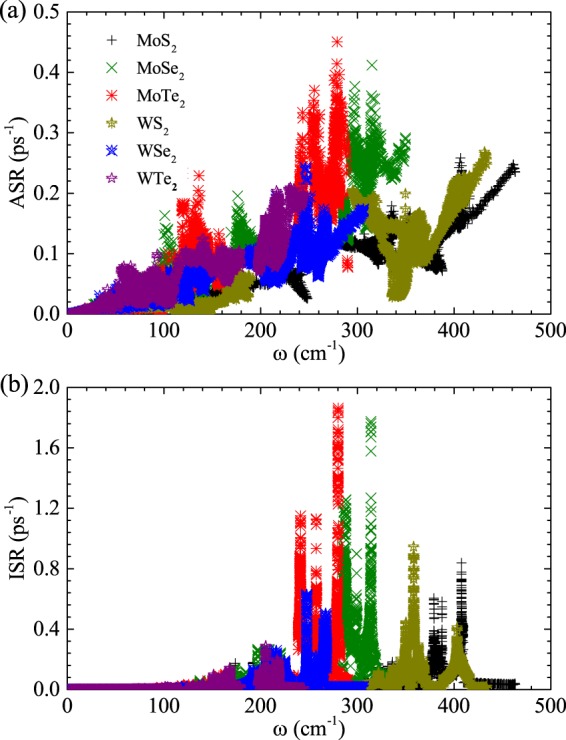
(**a**) Anharmonic three-phonon scattering rates (ASRs) (**b**) and isotopic scattering rates (ISRs) as functions of frequency for TMDC MX_2_ monolayers.

Keeping in view of the substantial difference in the contribution made by acoustic phonon modes towards the *κ* in TMDC MX_2_ monolayers and other 2D graphene like materials, it seems to be very meaningful to consider the relaxation time for each phonon mode as a function of frequency, as presented in Fig. 4. To avoid the boundary scattering effect in ShengBTE calculation procedures, the relation $1/{\tau }_{\lambda }=\mathrm{1/}{\tau }_{\lambda }^{anh}+1/{\tau }_{\lambda }^{iso}$, which is measured from the anharmonic three-phonon scattering and isotopic scattering, can be adopted to evaluate the total relaxation time in RTA. The summation of three-phonon transition probabilities ${{\rm{\Gamma }}}_{\lambda \lambda {\prime} \lambda {\prime} {\prime} }^{\pm }$ results into $\mathrm{1/}{\tau }_{\lambda }^{anh}$, which can directly be found from the anharmonic IFC3^45,46^. To understand the low thermal conductivity of the WTe_2_ monolayer, we have calculated the anharmonic three-phonon scattering and isotopic scattering rates for all TMDC MX_2_ monolayers. The calculated relaxation times behave differently for all TMDC *MX*_2_ monolayers. Figure 4 shows that in all cases, the acoustic modes relaxation times are shorter than that of optical modes. But this feature is surprisingly more dominating in case of the WTe_2_ monolayer, especially. It is well established fact that major contribution to *κ*_*L*_ mainly comes from the acoustic modes. Thus, as compared to the rest of the TMDC MX_2_ monolayers, the relaxation times for WTe_2_ of the acoustic modes are fairly short, which clearly indicates very strong phonon scattering in the WTe_2_ monolayer. Moreover, further comparison indicates that for most of the TA, LA, and LO modes, the relaxation time for the WTe_2_ monolayer is shorter than the rest of the family of TMDC MX_2_ monolayers because the TO modes of them retains longer relaxation time.

The isotopic relaxation time is also shown in Fig. 4(b). Isotopic scattering processes are sensitive to wavelength. Thus, the phonons of long-wavelength can transfer all the heat with small isotopic scattering^45,46^. Thus at low frequencies, the relative longer isotopic relaxation time of acoustic phonons can be observed. The inverse of the total relaxation time is a sum of contributions from anharmonic three-phonon scattering and isotopic scattering. For WTe_2_ monolayer, the span of the phonon frequency is much shorter than rest of MX_2_ monolayers. Figure 4(a,b) also show that for the WTe_2_ monolayer, the separation between acoustic and the optical phonon branches is much shorter than rest of the systems, which results in much more frequent scattering between acoustic modes and optical modes. In addition, the strength of such scatterings is expected to be strong compared with the rest of *MX*_2_ monolayers. The strong scattering in WTe_2_ monolayer is also correlated to their relatively small range of the phonon frequency. Thus, unlike the high thermal conductivity calculated in the case of rest of TMDC MX_2_ monolayers, the lattice thermal conductivities of WTe_2_ monolayer are found to be much lower, i.e. *κ*_*L*_ (33.66 Wm^−1^ K^−1^ at 300 K) when the size of sample is 1 *μm*, as shown in Fig. 2.

**Figure 5:**
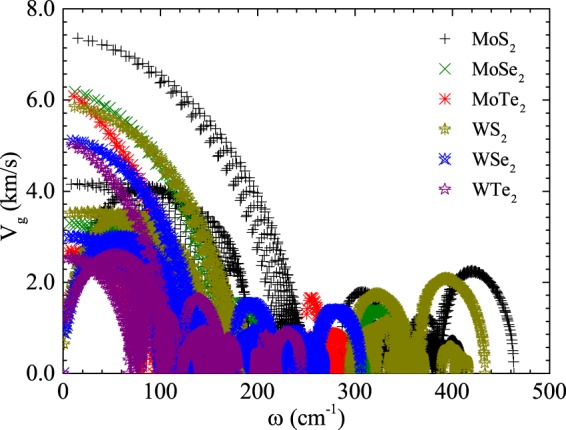
Phonon group velocities for all phonon modes within the first Brillouin zone as a function of frequency for TMDC MX_2_ monolayers.

Based on the lattice thermal conductivity equation (1), the value of lattice thermal conductivity is mainly determined by ASRs and the group velocities of phonons. The ASRs of WS_2_ is much lower than those of MoS_2_ as shown in Fig. 4(a), which interprets the higher thermal conductivity of WS_2_ than that of MoS_2_, although MoS_2_ possesses higher phonon group velocity. In detail, the ASRs derive from the sum of three-phonon transition probabilities, which are determined by the anharmonic IFCs and the weighted phase space. The strength of the anharmonic IFCs corresponds to the anharmonicity of phonon modes, which is usually characterized by the Grüneisen parameter (*γ*). The weighted phase space is a direct measure of the number of scattering processes. Our results show that the weighted phase spaces of MoS_2_ and WS_2_ are almost same to each other, as shown in Fig. 1S (see Supplementary Information). However, the Grüneisen parameter (*γ*) of MoS_2_ is higher than that of WS_2_, which means WS_2_ has a weak anharmonic IFCs, as shown in Fig. 2S (see Supplementary Information), and thus resulting in a lower ASRs and higher thermal conductivity. However, for MoSe_2_ and WSe_2_, their corresponding ASRs values are almost equivalent to each other as presented in Fig. 3S (see Supplementary Information). Thus, the difference in their group velocities mainly determines the existing difference in their thermal conductivities as mentioned in Table 1 (see Supplementary Information). As shown in Fig. 5, the calculated group velocities for MoSe_2_ are higher than those of WSe_2_, which mainly accounts for the higher thermal conductivity of MoSe_2_ than that of WSe_2_. Obviously, similar features prevail in case of MoTe_2_ and WTe_2_ as well, as shown in Figs 4 and 5, respectively.

### Phonon group velocities for all phonon modes

To get further insight to understand the difference of *κ*_*L*_ among this family of TMDC MX_2_ low dimensional materials, we have calculated the phonon group velocities that are extended over the first Brillouin zone as presented in Fig. 5. The equation *v*_*g*_ = *dω*_*n*_/*dq* is being used to find out the phonon group velocities for the *n*th branch regarding all the systems under consideration. As expected, the WTe_2_ monolayer which constitutes of the heavier atomic mass elements, possess much smaller group velocities than rest of the family of TMDC MX_2_ monolayers, particularly for the acoustic as well as optical branches. For one of the acoustic modes, the maximum group velocities for TMDC MX_2_ monolayers are about 7.35, 6.09, 6.08, 5.86, 5.03, and 5.02 km/s, respectively. But it is interesting to note that for those of optical modes, similar gradual decreasing trend in the group velocities is being observed as in the former cases of acoustic modes. Thus, for one of the optical modes, the maximum group velocities for TMDC *MX*_2_ monolayers are about 2.23, 2.06, 1.43, 1.65, 1.38, and 0.87 km/s, respectively. It can be seen the minimum group velocity for optical and the acoustic modes is only found in the case of the WT_2_ monolayer. These findings clearly support the lower *κ*_*L*_ that is proposed for WTe_2_ monolayer as determined for ${\kappa }_{L}\sim {v}_{g}^{2}$ using RTA. Hence, it is easy to analyze the dependence of *κ*_*L*_ on the polarization by combining the group velocities with relaxation time as shown in Fig. 2.

## Conclusion

In summary, we have systematically studied the thermal transport properties of monolayer transition metal dichalcogenides MX_2_ (M = Mo, W; X = S, Se, Te) using first-principles calculations and the Boltzmann transport equation for phonons. Our results indicate that monolayer WTe_2_ possesses the lowest lattice thermal conductivity *κ*_*L*_ (33.66 Wm^−1^ K^−1^ at 300 K) among this family of six semiconducting low dimensional materials, while the highest *κ*_*L*_ (113.97 Wm^−1^ K^−1^ at 300 K) comes from the WS_2_ monolayer. Further analyses reveal that the higher anharmonic and isotopic scatterings along lower phonon group velocities lead to the lowest obtained value of *κ*_*L*_ in case of WTe_2_. Additionally, we have also calculated the cumulative thermal conductivity *κ*_*C*_ as a function of mean free path, which suggests that the nanostructuring even up to the length of about 400 nm would reduce *κ*_*L*_ drastically. Keeping in view of the lowest thermal conductivity for the WTe_2_ monolayer, we propose that WTe_2_ monolayer may find its potential applications regarding the high-performance 2D nano-devices at nanoscale^47^.

## Supplementary information

LaTeX Supplementary File
