# Supplementary material for: Intrinsic Thermal conductivities of monolayer transition metal dichalcogenides MX2 (M = Mo, W; X = S, Se, Te)
Source: Sci Rep. 2019 Mar 14;9:4571. doi: 10.1038/s41598-019-40882-2 (PMC6418116; doi:10.1038/s41598-019-40882-2)
Supplement: Supplementary file 1 — LaTeX Supplementary File [file 41598_2019_40882_MOESM1_ESM.pdf]

Supplementary information for intrinsic thermal conductivities of monolayer transition metal dichalcogenides MX_2 (M=Mo, W; X=S, Se, Te)

Muhammad Zulfiqar[1,2,3,+], Yinchang Zhao[4,*], Geng Li[1,2,+], ZhengCao Li[5,+], and Jun Ni[1,2,*]

[1]State Key Laboratory of Low-Dimensional Quantum Physics, Department of Physics, Tsinghua University, Beijing 100084, People's Republic of China
[2]Collaborative Innovation Center of Quantum Matter, Beijing 100084, People's Republic of China
[3]Department of Physics, University of Sargodha, 40100 Sargodha, Pakistan
[4]Department of Physics, Yantai University, Yantai 264005, People's Republic of China
[*]Corresponding author: y.zhao@ytu.edu.cn
[5]Department of Material Science and Engineering, Tsinghua University, Beijing 100084, People's Republic of China
[*]Corresponding author: junni@mail.tsinghua.edu.cn

I. Comparison between the weighted phase space of MoS_2 and WS_2.

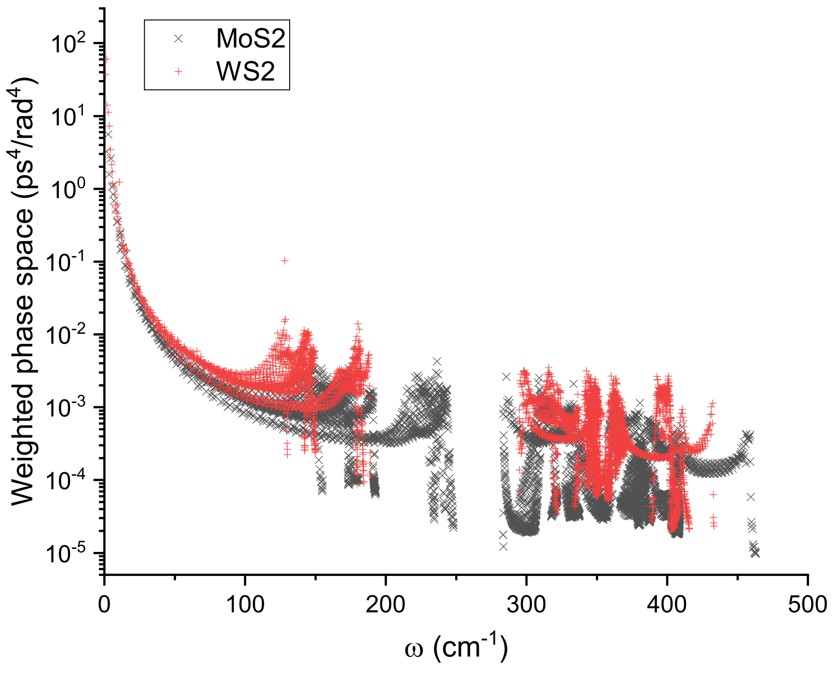

Figure 1S. (Color online) Weighted phase space W as a function of frequency for MoS_2 and WS_2 at 300K.

Based on the lattice thermal conductivity equation (1) in the main text, the value of lattice thermal conductivity is mainly determined by ASRs and the group velocities of phonons. The ASRs of WS_2 have much smaller values than those of other TMDC as shown in Fig.4(a), which accounts for the highest lattice

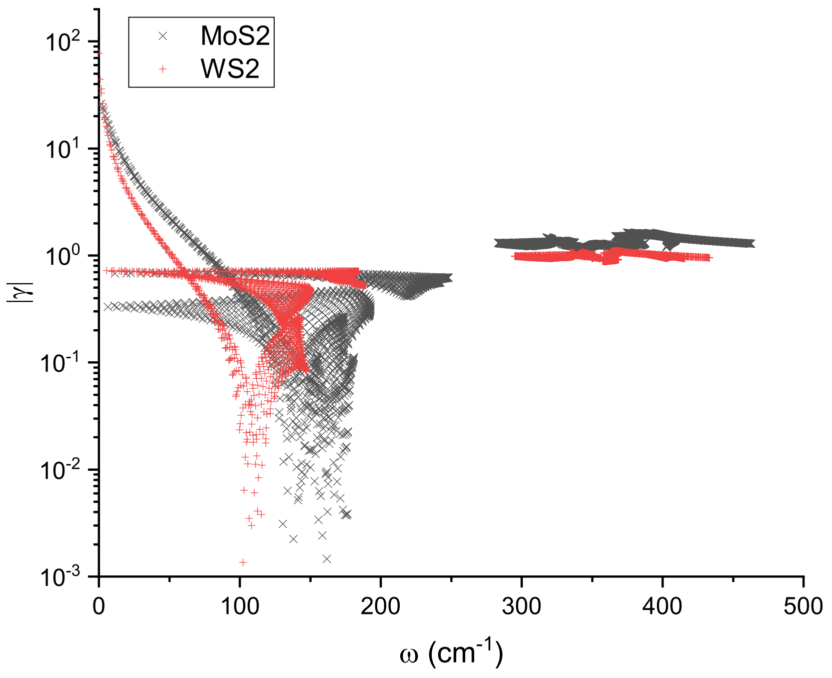

Figure 2S. (Color online) The Grüneisen parameter (γ) as a function of frequency for MoS_2 and WS_2 at 300K.

thermal conductivity although the phonon group velocities of WS_2 is smaller than those of MoS_2. In detail, the ASRs are determined by the anharmonic IFC3 and the weighted phase space. Our calculations show that the weighted phase space of WS_2 is higher than that of MoS_2, as shown in Fig. 1S. The higher weighted phase space corresponds to more three-phonon scattering channels, which seems to be contradictory with the lower ASRs of WS_2. To explain this, we calculate the lattice thermal conductivity of a hypothetical MoS_2 (WS_2), whose IFC3 is replaced by that of WS_2 (MoS_2), while all the other quantities remain the same. The result is 85.95 (95.32) $Wm^{-1}K^{-1}$ for the hypothetical WS_2 (MoS_2) at 300 K, in sharp contrast to 118.50 (73.38) $Wm^{-1}K^{-1}$ for the genuine one at the same temperature. This means that the IFC3 of MoS_2 is much stronger than that of WS_2, which is the main reason for the difference of anharmonicity between WS_2 and MoS_2.

However, the calculated value of Grüneisen parameter (γ) for MoS_2 is higher than WS_2, which suggests weak anharmonic IFCs for WS_2 as presented in Fig. 2S. It lowers ASRs and results into higher thermal conductivity.

II. Effect of group velocities difference on thermal conductivities.

The acoustic and optical phonons group velocities plays very important role regarding the obtained thermal conductivities for MX_2 monolayers. The difference between the maximum group velocities for acoustic and optical phonon modes are provided in Table 1. From Table 1, it can be seen that for all respective cases, the group velocities of acoustic phonon modes (at corresponding low frequency which mainly occurs due to unique heavy atom (Mo/W) present in the every unit cell) dominate instead of optical phonon modes (at corresponding high frequency which mainly occurs due to unique light atom (S/Se/Te) present in the every unit cell). Thus, we can establish the fact that for almost equivalent ASRs as shown in Fig. 3S, the difference in their group velocities mainly determines the existing difference in their thermal conductivities.

MX_2 monolayers	Group velocities (Km s^{-1}) for		Difference in group velocities (Km s^{-1})
	acoustic phonons	optical phonons	
MoS_2	7.4	2.3	5.1
$MoSe_2$	6.0	1.3	4.7
$MoTe_2$	6.1	1.6	4.4
WS_2	5.9	2.1	3.8
WSe_2	5.1	1.4	3.7
WTe_2	4.3	0.8	3.5

Table 1. Details for the calculated group velocities for MX_2 monolayers at their particular corresponding frequencies.

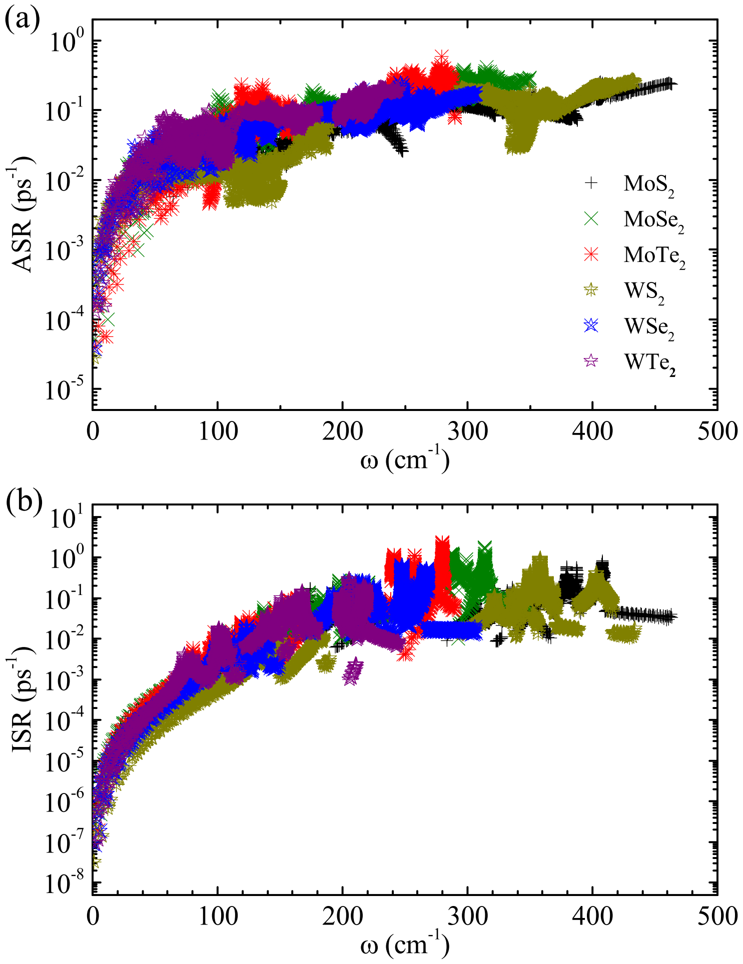

Figure 3S. (Color online) (a) More detailed anharmonic three-phonon scattering rates (ASRs) (b) and isotopic scattering rates (ISRs) including acoustic and phonon modes as function of frequency for TMDC MX_2 monolayers.
